# Latent Trajectories of Haematological, Hepatic, and Renal Profiles after Oil Spill Exposure: A Longitudinal Analysis

**DOI:** 10.3390/ijerph20042871

**Published:** 2023-02-06

**Authors:** Benjamin Atta Owusu, Apiradee Lim, Nitinun Pongsiri, Chanthip Intawong, Sunthorn Rheanpumikankit, Saijit Suksri, Thammasin Ingviya

**Affiliations:** 1 Department of Mathematics and Computer Science, Faculty of Science and Technology, Prince of Songkla University, Pattani Campus, Pattani 94000, Thailand; 2 Multidisciplinary Research and Innovation Centre, Kumasi AOK569, Ghana; 3 Air Pollution and Health Effect Research Center, Prince of Songkla University, Hat Yai Campus, Songkhla 90110, Thailand; 4 Division of Digital Innovation and Data Analytics, Faculty of Medicine, Prince of Songkla University, Hat Yai Campus, Songkhla 90110, Thailand; 5 Occupational Medicine Department, Rayong Hospital, Rayong 21000, Thailand; 6 Rayong Provincial Public Health Office, Rayong 21000, Thailand; 7 Department of Family and Preventive Medicine, Faculty of Medicine, Prince of Songkla University, Hat Yai Campus, Songkhla 90110, Thailand

**Keywords:** longitudinal trajectories, haematological profile, kidney function, liver function, latent classes

## Abstract

Exposure to polycyclic aromatic hydrocarbons (PAHs) and volatile organic compounds (VOCs) in crude oil has carcinogenic effects on various organ systems. This longitudinal cohort study examined the effects of oil spill exposure on the haematological, hepatic, and renal profiles of Rayong oil spill clean-up workers. The sample included 869 clean-up workers from the Rayong oil spill. Latent class mixture models were used to investigate and classify the longitudinal trajectories and trends of the haematological, hepatic, and renal indices. Subgroup analysis was used to evaluate the association between the urinary metabolites of PAHs and VOCs and haematological, hepatic, and renal parameters. Most clean-up workers (97.6%) had increasing levels of white blood cells (WBCs) (0.03 × 10^3^ cells/µL), 94.90% of the workers had a significantly increasing trend of blood urea nitrogen (0.31 mg/dL per year), and 87.20% had a significantly increasing trend of serum creatinine (0.01 mg/dL per year). A high–decreasing trend of WBCs was seen in 2.42% (−0.73 × 10^3^ per year). Post-exposure changes in haematological, renal, and hepatic profiles are present in workers exposed to the Rayong oil spill. This indicates possible long-term health complications and worsening renal function after exposure to PAHs and VOCs in crude oil.

## 1. Introduction

Oil spill disasters have released toxic chemicals, such as polycyclic aromatic hydrocarbons (PAHs) and volatile organic compounds (VOCs), into marine and terrestrial ecosystems for decades following the invention of liquid propellants [1,2]. In most oil spills, local citizens voluntarily participate in clean-up activities and become exposed to various levels of toxicants. PAHs and VOCs in crude oil, such as benzo[a]pyrene, are classified as Group I human carcinogens by the International Agency for Research on Cancer [3,4]. The intake of these compounds, primarily by inhalation, may induce severe adverse health effects, including alterations in haematological, hepatic, and renal functions [5,6].

The quantification of urinary metabolites of PAH and VOCs to estimate exposure levels has been investigated previously [7,8]. The levels of 1-hydroxypyrene-glucuronide (1-OHPG) and trans,trans-muconic acid (t,t-MA) have been widely used to assess environmental exposure to PAHs [7,8] and VOCs (e.g., benzene), respectively [9,10,11]. The levels of urine metabolites represent quantifiable levels of exposure from multiple routes and sources, for instance, cigarette smoke and oil spills [12,13].

The complete blood count (CBC) and serum levels of renal and liver biomarkers are commonly used to assess the effects of crude oil exposure in humans and other organisms. Several studies have used haematological, hepatic, and renal parameters to assess the effects of exposure to PAH and VOC on human health [6,14,15,16]. However, the effects on these parameters vary according to the level and duration of exposure, as well as by the influence of other environmental factors.

The Rayong oil spill of 2013 was caused by a ruptured pipeline, whereby more than 50,000 L of crude oil spilled into the Gulf of Thailand. Until January 2022, this was the most recent marine disaster in Thailand. An observational baseline study conducted by Ingviya et al. [17] assessed urinary 1-OHPG and t,t-MA concentrations to determine the internal exposure to PAHs and VOCs among clean-up workers of the Rayong oil spill on different clean-up days. The study showed that workers who participated during the initial days of clean-up had significantly higher exposure, as indicated by higher levels of 1-OHPG, while the t,t-MA concentration was undetectable in the majority of clean-up workers throughout the clean-up period [17]. Similarly, in an observational study, Owusu et al. [18] assessed endpoint changes in haematological, hepatic, and renal indices among 570 Rayong oil spill clean-up workers. The exposure levels were differentiated based on their work on different clean-up days.

In this study, we hypothesised that exposure to the Rayong oil spill induced different nonlinear trajectories of haematological, hepatic, and renal profiles. We aimed to examine the long-term effects of toxicants (PAHs and VOCs) by assessing the 5-year trajectories of haematological, hepatic, and renal indices after the 2013 Rayong oil spill. Primarily, we examined the presence of distinct longitudinal trajectories of these indices among clean-up workers and their associated factors, including age. Finally, long-term changes in the haematological, hepatic, and renal indices by the exposure levels of PAHs and VOCs were assessed using urinary concentrations of the toxicant metabolites. Assessing these trajectories can guide and encourage the implementation of health follow-up protocols for workers who clean oil spills.

## 2. Materials and Methods

### 2.1. Study Design and Setting

In this retrospective longitudinal cohort study, we analysed haematological, liver, and kidney function parameters, measured at baseline and during a 5-year follow-up after the 2013 Rayong oil spill. The exposure data of the cohort were retrieved from the baseline post-shift urinary concentrations of 1-OHPG and t,t-MA, quantified by Ingviya et al. [17]. Data were retrieved from the record forms initially collected by Rayong Hospital as part of health services.

### 2.2. Ethics Approval and Informed Consent

Clean-up workers verbally consented to the use of their data for further analysis to improve the quality of health services and follow-up protocols. 

### 2.3. Study Population and Measurements

During and after the Rayong oil spill clean-up activities, the Rayong Provincial Health Office collaborated with Rayong Hospital to undertake a 5-year health surveillance of clean-up workers. After the oil spill, the workers were invited to undergo annual health assessments, including laboratory tests, CBCs, and hepatic and renal function parameters. The dataset included 869 Rayong oil spill clean-up workers that attended at least one follow-up visit between 2014 and 2018. A subgroup analysis was conducted on 169 workers who provided urine samples to determine 1-OHPG and t,t-MA urinary concentrations. Demographic information including age, sex, and background occupation was collected. Urinary cotinine levels were measured, and a cut-off of 50 ng/mL used to differentiate between non-smokers and smokers [19]. Data on specific clean-up activities were also collected. Haematological, hepatic, and renal parameters at baseline and follow-up were retrieved from the electronic records of the Rayong Provincial Health Office and Rayong Hospital.

### 2.4. Quantification of 1-OHPG and t,t-MA Concentrations

The concentrations of 1-OHPG and t,t-MA were quantified in urine samples collected during clean-up in 2013. Immunoaffinity chromatography and synchronous fluorescence spectroscopy were used to measure the 1-OHPG concentration at Paul Strickland laboratory at John Hopkins University (limit of detection 0.04 pmol/mL; coefficient of variation 5.6%) [17]. Baseline 1-OHPG concentration was categorised as high (>5.0 pmol/mL), moderate (1.0–5.0 pmol/mL), or low (<1.0 pmol/mL), based on a study by Kang et al. [20].

Urinary t,t-MA concentration was analysed using high-performance liquid chromatography with fluorescence detection in three laboratories, including Rayong Hospital and two other private laboratories. The agreement between the t,t-MA measurements reported by the three laboratories was 99.99% [21]. The t,t-MA concentration was classified as detectable or undetectable because a large proportion of the samples had levels below the limit of detection (0.01 mg/dL) of the assay.

### 2.5. Measurement of Haematological, Hepatic, and Renal Parameters

Approved medical protocols in a standardised laboratory at the Royal Hospital were used for analysis. The CBC parameters, including haemoglobin, haematocrit, red blood cell count (RBC), white blood cell count (WBC), absolute neutrophil count (ANC), and platelet count, were measured using an automatic CBC analyser. Hepatic [serum aspartate transaminase (AST) and alanine transaminase (ALT)] and renal indices [blood urea nitrogen (BUN) and serum creatinine (Cr)] were determined using enzymatic assay techniques.

### 2.6. Statistical Analysis

Descriptive statistics (mean, standard deviation, and percentages) were used for demographic data of the oil spill clean-up workers and to summarise the haematological and hepatic profiles. Generalised mixed-effects models accounted for the expected within-subject correlation between the repeated measures. Latent class mixture models (LCMMs) were used to investigate and classify the longitudinal trajectories and trends of the haematological, hepatic, and renal indices, while adjusting for age [22,23,24]. The “lcmm” package in R was used for the latent class trajectory analysis [25]. Polynomial specifications as functions of time, with a number of latent classes ranging from one to three, were used in the LCMMs to assess the different trends. The optimal number of latent classes for each model was determined using the Bayesian information criterion (BIC) and posterior probabilities above a 0.7 threshold [25]. The latent classes for each haematological, hepatic, and renal index were labelled using the observed mean at baseline (high, low, and normal) and their adjusted trends during the study (stable, increasing, and decreasing). The definitions of high, low, and normal concentrations were based on the standard medical reference ranges for each index at baseline [26]. We examined the yearly class-specific observed and predicted means of each haematological, hepatic, and renal index. Furthermore, we examined the effects of age and the latent longitudinal trajectories of the indices by assessing age-specific trajectories.

A subgroup analysis of 169 oil spill clean-up workers was performed to determine the effects of baseline urinary concentrations of 1-OHPG and t,t-MA and smoking status on the overall trajectories and trends of haematological and hepatorenal profiles. Age-centred generalised linear mixed models were used for the subgroup analysis. The characteristics of the subgroup, including age, gender, and background occupation, were compared to the characteristics of the full cohort (869 workers) to assess the possibility of selection bias. A two-sample t-test was used to test the hypothesis that the average age of the full cohort was equal to the average age of the subgroup. Similarly, a Chi-square test with Yates’ continuity correction was used to test the proportion of gender and background occupation of the subgroup and the full cohort. All statistical analyses and graphical displays were performed using R version 4.0.5 [27].

## 3. Results

### 3.1. Demographics of All Oil Spill Clean-Up Workers (n = 869)

During the clean-up, 2118 workers engaged in various activities. The demographic characteristics of these workers have been reported previously [9,17]. During the 5-year follow-up surveillance, 869 clean-up workers reported to Rayong Hospital at least once. The demographics of the 869 participants are summarised in Table 1.

The mean (±SD) age of the clean-up workers at baseline was 39.47 ± 10.23 years, and 86.30% of the study sample was male. Civilian volunteers and PTT global chemical (PTTGC) staff accounted for 29.80% and 38.80% of all clean-up workers, respectively. Based on the cotinine levels, 109 (12.50%) workers were active smokers (>50 ng/mL). Demographic characteristics and baseline concentrations of 1-OHPG and t,t-MA are summarised in Table 1.

### 3.2. Characteristics of Subgroup of Oil Spill Clean-Up Workers (n = 169)

A total of 169 oil spill clean-up workers provided post-shift urine samples and reported at least one follow-up visit. The demographic characteristics of the 169 workers are summarised in Table 2. The mean (±SD) age was 39.72 ± 9.91 years and 94.70% were men. Civilian volunteers accounted for 44.40% of the 169 workers. At baseline post-shift, 17 (10.10%) clean-up workers had high levels of 1-OHPG (>5.0 pmol/mol), 55 (32.50%) had moderate levels (1.00–5.00 pmol/mL), and 97 (57.40%) had low levels (<1.0 pmol/mol). Overall, 48 (28.40%) workers had detectable urinary t,t-MA levels. The demographic characteristics and baseline concentrations of 1-OHPG and t,t-MA in the 169 workers are summarised in Table 2.

Comparing between the subgroup and full cohort of 869 workers, the mean ages were not significantly different (Supplementary Table S1). However, the characteristics of the subgroup and the whole cohort were different in terms of gender and background occupation (Supplementary Table S1).

### 3.3. Latent Trajectories and Trends of Haematological, Hepatic, and Renal Parameters (n = 869)

The LCMMs were used to classify the trends of haematological, hepatic, and renal parameters of all 869 participants. The analyses showed that Rayong oil spill clean-up workers had between one and four latent trends for each index.

**Figure 1:**
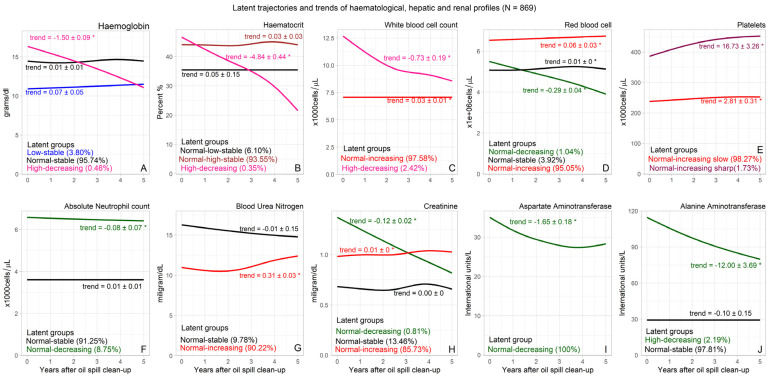
Latent trends of the haematological and hepatic parameters. (**A**) Haemoblogin: Only 3 significant latent groups: Low-stable, Normal-stable, high-decreasing. (**B**) Haematocrit: Only 3 significant latent groups: Normal-low-stable, Normal-high-stable, High-decreasing. (**C**) White blood cell: Only 2 significant latent groups: Normal-increasing, Normal-decreasing. (**D**) Red blood cell: Only 3 significant latent groups: Normal-decreasing, Normal-stable, Normal-increasing. (**E**) Platelet counts: Only 2 significant latent groups: Normal-increasing slow, Normal-increasing sharp. (**F**) Absolute Neutrophil count: Two significant latent groups: Normal-stable, Normal-decreasing. (**G**) Blood urea nitrogen: Only 2 significant latent groups: Normal-stable, Normal-increasing. (**H**) Serum creatinine: Only 3 significant latent groups: Normal-decreasing, Normal-stable, Normal-increasing. (**I**) Aspartate aminotransferase: Only 1 latent group: Normal-decreasing. (**J**) aminotransferase: Only 2 significant latent groups: Normal-decreasing, Normal-stable Note: (*) represents significant trends.

The best LCMM (based on BIC and posterior probabilities) for haemoglobin had three latent groups (high-, low-, and normal-stable). There were 33 clean-up workers (3.80%) with low haemoglobin levels at baseline and a stable trend over time (low-stable). Four workers (0.46%) had high haemoglobin levels at baseline, but with a significantly decreasing trend of 1.50 g/dL per year. At the baseline, the low-stable latent group had observed and predicted mean haemoglobin levels of 10.9 g/dL and 11.1 g/dL, respectively. The observed and predicted mean haemoglobin levels for the low-stable class were relatively stable over the period of the study, with no significant differences over time. The observed mean haemoglobin level for the high-decreasing group ranged between 16.8 g/dL at the baseline and 9.22 g/dL in 2018, while the predicted level ranged from 16.9 g/dL to 9.74 g/dL. The clean-up workers had three latent groups based on the levels and trends of haematocrit. Average haematocrit percentage was high among three (0.35%) clean-up workers, with a decreasing trend (4.84% per year). The remaining 866 (99.65) workers had normal haematocrit percentage at baseline, of which 6.10% had relatively lower average haematocrit percentage (indicated with asterisks), compared to 90.60%. Among the workers with high decreasing trend, the observed mean haematocrit percent was 46.9% at baseline, while the mean percent at the fifth year was 17%. The observed and predicted mean haematocrit levels for most of the workers were almost the same for every year. The WBC count of 2.42% of the clean-up workers demonstrated a high–decreasing trend (−0.73 × 10^3^ cells/µL per year). Among these workers, the observed mean WBC count reduced from 12.79 × 10^3^ cells/µL at baseline to 8.00 × 10^3^ cells/µL in 2018. The mean WBC count for 97.5% of the workers increased slightly from 7.05 × 10^3^ cells/µL to 7.11 × 10^3^ cells/µL. All oil spill clean-up workers had normal RBC, platelet, and ANC levels at baseline. The majority of the workers (95.00%) had increasing trends of RBC from an average of 5.08 cells/µL to 5.12 cells/µL (trend = 0.01 cells/µL per year), and 8.75% had decreasing trends of ANC over time (−0.08 × 10^3^ cells/µL per year) (Figure 1).

**Figure 2:**
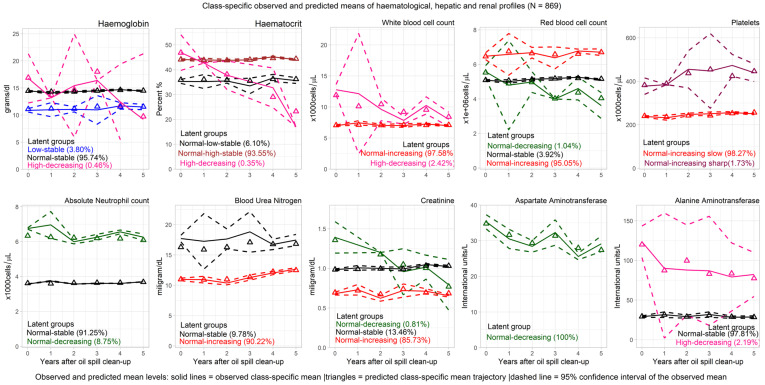
Class-specific observed and predicted means of haematological, hepatic, and renal profiles.

The average BUN level grouped the Rayong oil clean-up workers into two latent classes (normal–increasing and normal–stable), whereas three latent groups were observed for Cr levels (normal–decreasing, normal–stable, and normal-increasing). Although the average BUN and Cr levels were normal at baseline for all clean-up workers, 90.20% of the workers had a significantly increasing trend of BUN (0.31 mg/dL per year), and 85.70% had a significantly increasing trend of Cr (0.01 mg/dL per year). The observed mean BUN level for the normal–increasing group was 10.97 mg/dL at the baseline, and 12.65 mg/dL at the fifth year of the follow-up. Meanwhile, the Cr level increased from 0.98 mg/dL to 1.02 mg/dL among the normal increasing group. The latent trajectories and trends of haematological, hepatic, and renal parameters for all oil spill clean-up workers are shown in Figure 1, while the observed and predicted class-specific means for each haematological, hepatic, and renal index are shown in Figure 2.

### 3.4. Longitudinal Trajectories by Age Group

The trajectories of haemoglobin, haematocrit WBC, RBC, and platelets were similar for all age groups; however, the levels of these parameters at baseline were lower for the age group of 50 years and older. These parameters showed a slightly increasing trend over time, which did not differ by age.

The average BUN at baseline ranged between 10.5–12.5 mg/dL among all the workers. Average BUN and Cr levels increased throughout the study period. These trajectories were consistent for all age groups, except for those aged 50 years and above, whose BUN and Cr increased sharply throughout the study period.

**Figure 3:**
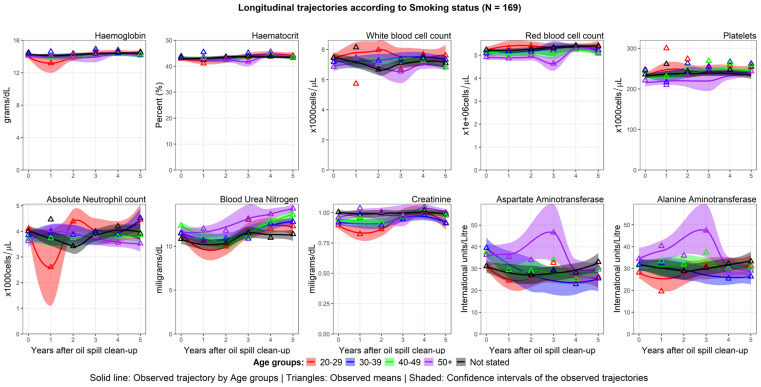
Longitudinal trajectories by age group at baseline.

AST and ALT levels showed stable trajectories among clean-up workers aged <50 years. Among workers older than 50 years, AST and ALT had an N-shaped trajectory, reaching peak levels three years after the clean-up work. The longitudinal trajectories of haematological, hepatic, and renal parameters according to age group are shown in Figure 3.

Although the RBC, WBC, and ANC show multiple slope trajectories, the variations between the baseline and the final follow-up were still within the normal range. These trajectories could be within normal biological variation.

### 3.5. Longitudinal Trajectories by Smoking Status (n = 169)

The longitudinal trajectories of haematological, hepatic, and renal parameters varied significantly according to smoking status. The average haemoglobin and haematocrit levels in non-smokers exhibited a decreasing trend over the study period, whereas the levels fluctuated in smokers.

**Figure 4:**
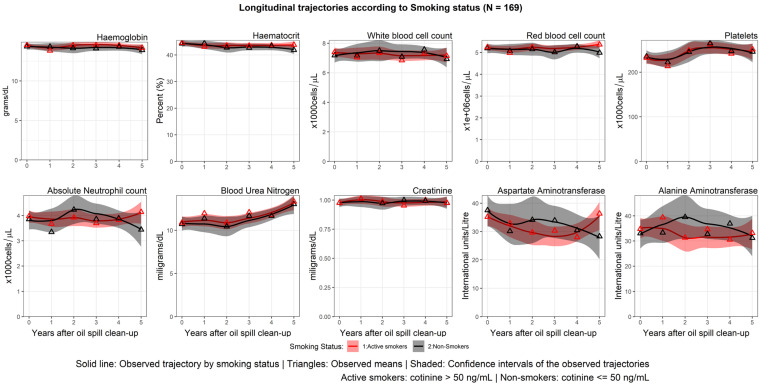
Longitudinal trajectories by smoking status.

The average WBC and ANC levels among non-smokers showed an N-shaped trajectory during the 5 years after the clean-up. Meanwhile, the WBC and ANC counts from smokers had a decreasing trajectory, particularly between the third and fourth years. The trajectories of BUN were stable during the 2 years and showed an increasing trend at 3–5 years after the clean-up for both smokers and non-smokers. The Cr, AST, and ALT levels of smokers and non-smokers fluctuated during 5 years of follow-up. The longitudinal trajectories of haematological, hepatic, and renal parameters by smoking status of the 169 oil spill clean-up workers are shown in Figure 4.

### 3.6. Longitudinal Trajectories by Concentrations of Urinary 1-OHPG and t,t-MA (n = 169)

**Figure 5:**
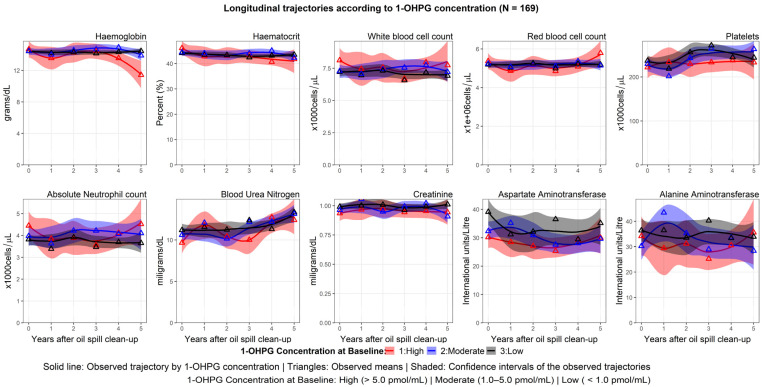
Longitudinal trajectories of the haematological, hepatic, and renal indices by the baseline concentration of 1-OHPG.

Most haematological indices showed varying trajectories. Haemoglobin and haematocrit levels among workers with high urinary 1-OHPG concentrations exhibited a sigmoid pattern with decreasing levels over time compared with the other exposure groups. Workers with low urinary 1-OHPG concentrations had stable trajectories of haemoglobin and haematocrit levels over time. The trajectories of WBC, ANC, and platelets were almost linearly stable during the five years, except workers with high urinary 1-OHPG concentrations exhibited a relatively lower platelet count than those in the other exposure groups. Figure 5 depicts the trajectories of all the indices based on urinary 1-OHPG concentrations.

**Figure 6:**
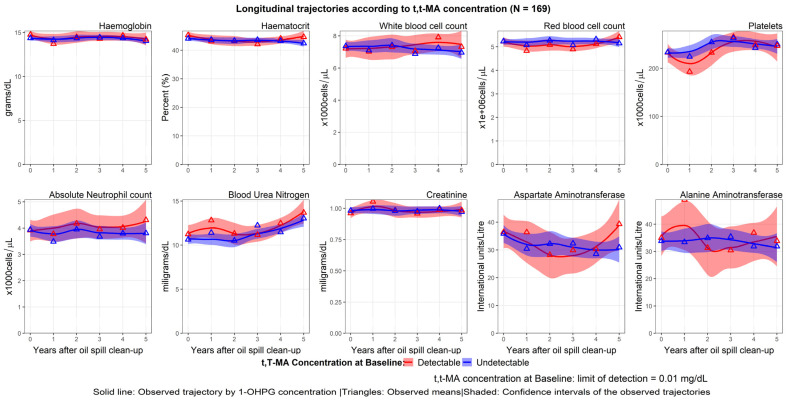
Longitudinal trajectories of the haematological, hepatic, and renal indices by the baseline concentration of t,t-MA.

Urinary t,t-MA was not detectable in 71.6% of the workers. The longitudinal trajectories of workers with detectable and undetectable concentrations of t,t-MA differed for most indices. The haematocrit level for a detectable t,t-MA concentration showed a U-shaped trajectory and decreased gradually until the fourth follow-up. WBC and ANC counts were generally higher in the detectable group than in the undetectable group. The longitudinal trajectories of the platelet counts were similar for both t,t-MA groups. The trends of BUN and Cr levels did not visibly differ between the detectable and undetectable groups. The longitudinal trajectories of the haematological, hepatic, and renal indices according to the baseline t,t-MA concentrations are shown in Figure 6.

## 4. Discussion

This longitudinal cohort study identified different latent longitudinal trajectories and trends in the haematological, hepatic, and renal profiles of 869 Rayong oil spill clean-up workers. Linking exposure to PAHs and VOCs, subgroup analyses assessed the longitudinal trend of the profiles among 169 cleaners with available data for urinary concentrations of 1-OHPG and t,t-MA.

Generally, no significant changes in haemoglobin and haematocrit levels were found among most clean-up workers, except for an observable decreased haemoglobin (<10 g/dL) and haematocrit (<24%) among a small group of workers. These workers might develop anaemic symptoms, such as fatigue, syncope, or lower work tolerance [28]. Age and cigarette smoking at the time of the oil spill clean-up showed minimal to no effects on haemoglobin and haematocrit levels. Subgroup analysis indicated that high 1-OHPG and detectable t,t-MA levels might be associated with reduced haemoglobin and haematocrit levels at baseline and with decreasing trajectories over time. Acutely elevated haemoglobin has been reported among a Canadian sub-population exposed to VOCs [28], which is consistent with the high haemoglobin level observed among a fraction of Rayong oil spill clean-up workers. Some studies conducted 2–7 years after oil spill exposure reported no significant changes in haemoglobin levels [5,6]. Studies have found that PAHs and VOCs can induce haemolytic anaemia [29,30], which may result in reduced haemoglobin and haematocrit levels. Another study by Kamal et al., also reported that exposure to PAHs is associated with reduced haematocrit levels in humans [15]. Consistently, our subgroup analysis revealed that urinary PAH and VOC metabolite levels were associated with reduced haemoglobin and haematocrit levels. Although chronic smoking is known to increase haemoglobin and haematocrit levels in human and animal models in response to hypoxaemia [31,32], this study cohort of smokers exhibited decreased or stable haemoglobin and haematocrit levels during the follow-up period. These findings suggest that the longitudinal changes in haemoglobin and haematocrit among clean-up workers are likely due to exposure to PAH and VOCs from other sources, including crude oil, rather than cigarette smoking.

Two remarkable trends in WBC, RBC, and platelet counts should be noted in workers. These haematological indices exhibited reduced levels and decreasing trends over time in a small group of workers, while some workers exhibited increasing trajectories in these indices. From the trajectory analysis, both trends were not associated with age and were only partially associated with smoking. Workers with high and detectable concentrations of 1-OHPG and t,t-MA at baseline had higher trajectories of WBC, RBC, and platelet counts than those with low or undetectable concentrations.

Increased WBC, RBC, and platelet counts due to oil spill exposure have been reported in previous studies [6,33]. A population-based study reported that high concentrations of VOC and PAH metabolites were associated with elevated levels of haematological indices, including WBC and RBC [28]. Similarly, the concentrations of 1-OHPG and t,t-MA were associated with high WBC and RBC counts in Rayong oil spill workers. Following exposure to oil spills and xenobiotic metabolism, benzene-active metabolites, including tt-muconaldehyde, 1,4-benzoquinone, hydroquinone, and catechol, can suppress the bone marrow [34,35,36]. Such haematopoietic damage can manifest in many ways, including alterations in the haematological profiles. Therefore, the alterations in haematological functions identified among the Rayong oil spill clean-up workers may be attributable to the suppression of bone marrow function by the effects of PAHs and VOCs in crude oil.

All oil-spill clean-up workers had normal BUN and Cr levels at baseline, but most had corresponding increasing trends. The longitudinal trajectories of the kidney indices were age-related, as higher BUN and Cr levels were observed among workers in the older age group. Higher and moderate 1-OHPG and detectable t,t-MA concentrations correlated with increasing BUN levels. Despite the observable association between BUN, Cr, and age [37], the possible effects of oil spill exposure on renal indices cannot be ruled out [5,6]. PAH metabolites, such as 1-OHPG, can cause oxidative stress and induce kidney damage [38,39]. AST and ALT levels are surrogates for hepatocellular injury and are highly sensitive to PAH and VOC exposure, as reported in various studies [40,41]. Although studies have reported possible liver damage from exposure to PAHs and VOCs, longitudinal trends in our research indicated no significant changes.

### Strengths and Limitations of the Study

This study found possible alterations in the haematopoietic system of oil spill clean-up workers. The findings of this study should be interpreted with consideration of its limitations. Most clean-up workers who attended follow-up visits did not provide urine samples at the baseline. The information on the concentrations of 1-OHPG and t,t-MA to assess exposure levels was only available for a relatively small group of workers. Nonetheless, the characteristics of this subgroup are similar to those of the entire cohort. Therefore, our results may be generalisable to the entire cohort. Additionally, selection (volunteering) bias was possible because the follow-up visit was voluntary. The characteristics of the workers who provided urine samples were different from that of the full cohort of 869 workers. Therefore, results from analysis based on urine biomarker concentration should be interpreted cautiously. Although most of the significant trends are shown in the small groups, with relatively small sample size, the trends are clinically significant. For instance, for haematocrit, the level decreases until possible anaemic symptoms could occur. Despite these limitations, this study provides insight into the possible long-term effects of oil spill exposure.

Previous studies involving subjects exposed to oil spill exposure have reported general findings. However, the latent class trajectory analysis used in this study identified small unique groups of oil spill clean-up workers with significant changes in haematological, hepatic, or renal profiles. Our findings suggest possible long-term haematopoietic function alterations from oil spill exposure, indicated by non-linear trajectories and significant long-term changes in haematological, hepatic, and renal profiles in workers after the oil spill. However, to assess whether these results were incidental or due to oil spill exposure, more studies including prospective cohort studies with a rigorous methodologic design and with periodic biomarker measurements for the oil spill clean-up workers are needed.

## 5. Conclusions

More than 5 years after exposure to PAHs and VOCs through oil spill clean-ups, alterations in the haematological, hepatic, and renal systems were still observable among the workers. Therefore, crude oil-related activities such as oil spill clean-up should be classified as potentially dangerous jobs exposing workers to carcinogens and haematotoxins. Personal protective equipment should be used in crude oil-related activities such as oil spill clean-up to reduce the risks of the exposure to carcinogens and haematotoxins. Health monitoring including blood sampling for haematological, hepatic, and renal indices should be planned before the workers participate in oil spill clean-ups to include assessments throughout the pre-placement of jobs and immediately post-shift, and then periodically for years following the clean-up. Future studies should assess the changes of biological indices adding oxidative stress biomarkers such as malondialdehyde and epigenetic changes, starting from pre-placement examination to further study the possibility of the acute and long-term effects from the oil spill.

## Figures and Tables

**Table 1:** Description of the participants’ demographics.

Demographic Factor	Description	Number of Workers	Percentage
Sex	Men	750	86.30%
	Women	119	13.70%
Age group at baseline	20–29	140	16.10%
	30–39	167	19.20%
	40–49	276	31.80%
	50+	93	10.70%
	Not stated	193	22.20%
Background occupation	* PTTGC staff	337	38.80%
	Civilian	259	29.80%
	Military	273	31.40%
Smoking status			
Non-smokers	Cotinine < 50 ng/mL	60	6.90%
Smokers	Cotinine > 50 ng/mL	109	12.50%
Unknown	Cotinine levels are not available.	700	80.60%

* PTT global chemical (PTTGC).

**Table 2:** Characteristics of the subgroup with available urine samples.

Demographic Factors	Description	Number of Workers	Percentage
Sex	Men	160	94.70%
	Women	9	5.30%
Age group at baseline (years)	20–29	22	13.00%
	30–39	40	23.90%
	40–49	73	43.10%
	50+	34	20.00%
Background occupation	PTTGC staff	29	17.20%
	Civilian	75	44.40%
	Military	65	38.40%
Cotinine level (ng/mL)	Median (1st–3rd quartiles)	7.51 (3.11–1040.79)	
Non-smokers	Cotinine < 50 ng/mL	109	64.50%
Smokers	Cotinine > 50 ng/mL	60	35.50%
* 1-OHPG (*n* = 169)	Median (1st–3rd quartiles)	0.76 (0.31–2.27)	
High	1-OHPG > 5.0 pmol/mL	17	10.10%
Low	1-OHPG < 1.0 pmol/mL	97	57.40%
Moderate	1.0–5.0 pmol/mL	55	32.50%
** t,t-MA (*n* = 169)	Median (1st–3rd quartiles)	0. 00 (0.00–36.40)	
	Detectable	48	28.40%
	Undetectable	121	71.60%

* 1-hydroxypyrene-glucuronide (1-OHPG); ** trans,trans-muconic acid (t,t-MA).

## Data Availability

The data, codes, and materials generated and analysed in this published article are available from the corresponding author upon reasonable request.

## Supplementary Materials

The following supporting information can be downloaded at: https://www.mdpi.com/article/10.3390/ijerph20042871/s1, Table S1: Comparison of demographic between the cohort (N = 869) and the subgroup (N = 169).
