# Supplementary material for: Latent Trajectories of Haematological, Hepatic, and Renal Profiles after Oil Spill Exposure: A Longitudinal Analysis
Source: Int J Environ Res Public Health. 2023 Feb 6;20(4):2871. doi: 10.3390/ijerph20042871 (PMC9956276; doi:10.3390/ijerph20042871)
Supplement: Supplementary file 1 [file ijerph-20-02871-s001.zip › ijerph-2089574-supplementary.pdf]

Table S1. Comparison of demographic between the cohort (N=869) and the subgroup (N = 169)

	Whole sample	Subgroup	Test	p-value
Size	869	169	t-test	
Age (mean)	39.5	39.7	t = -0.3,	0.8
Group (N)	Compare proportions with Chi-square		Chi-square value	< 0.05
Civilians	259	75	30	
Military	273	65		
PTTGC	337	29		
Gender (N)	Compare proportions with Chi-square		8	< 0.05
Female	119	9		
Male	750	160		
